# Nanovaccines in gastrointestinal cancers

**DOI:** 10.3389/fimmu.2025.1680053

**Published:** 2025-12-15

**Authors:** YuHan Wang, Peng Huang, Chun Li, ShengJin Tu, Hua Yang

**Affiliations:** 1 Department of General Surgery, Zigong Fourth People’s Hospital, Zigong, Sichuan, China; 2 Division of Abdominal Tumor Multimodality Treatment, Cancer Center, West China Hospital, Sichuan University, Chengdu, Sichuan, China

**Keywords:** gastrointestinal cancers, immunotherapy, nanomedicine, precision medicine, vaccine

## Abstract

Cancers of the gastrointestinal (GI) tract rank among the most commonly diagnosed malignancies worldwide, posing a heavy burden on public health. Therapeutic tumor vaccines have garnered significant interest due to their ability to promote tumor regression, eliminate minimal residual disease, create enduring immune memory, and minimize non-specific adverse effects. Recently, the integration of nanotechnology into cancer immunotherapy, particularly through the development of nanovaccines, represents a transformative approach to treating GI cancers. This review outlines the significant advancements in the design and application of nanovaccines, emphasizing the mechanisms by which these nanovaccines deliver tumor-specific antigens and immunostimulatory adjuvants, ensuring effective activation of immune responses. Despite the promise these innovative therapies hold, challenges remain, including efficient antigen delivery, safety concerns, and the complexities associated with regulatory compliance. This comprehensive analysis highlights the potential of nanovaccines in transforming treatment paradigms for GI cancers while underscoring the need for collaborative efforts to accelerate their clinical translation.

## 1 Introduction

Globally, cancers of the gastrointestinal (GI) tract are among the most frequently diagnosed malignancies, accounting for a substantial proportion of cancer cases in various populations (1). These cancers include a diverse range of tumors affecting different parts of the GI system, such as the esophagus, stomach, pancreas, liver, colon, and rectum (2). Due to their prevalence, GI tract cancers pose a significant public health challenge, contributing to high rates of morbidity and mortality worldwide (3, 4). The complex nature of these cancers, combined with diverse risk factors such as unhealthy diet, genetic predisposition, and environmental influences, complicates early detection and effective treatment (5–7). As a result, many patients present with advanced disease, which often leads to poorer outcomes and increased healthcare costs (8). Despite advancements in diagnostic techniques and treatment options, GI cancers continue to present a poor prognosis due to their genetic complexities, resistance to treatment, and tendency for metastasis (9, 10). Therefore, there is an urgent need to develop novel therapeutic strategy for combating this disease.

Cancer immunotherapy, utilizing the immune system to impede tumor growth, is increasingly viewed as a promising strategy for effectively treating and potentially curing certain cancer types (11). This strategy focuses on adjusting the immune system or employing immune cells to stimulate or enhance the immune system’s ability to recognize and eliminate cancer cells through natural processes that can be bypassed as the disease progresses (12, 13). Recent clinical trials, particularly those involving immune checkpoint inhibitors (ICIs) targeting immune checkpoint molecules have demonstrated significant efficacy in GI cancers and have contributed to a shift in treatment principles (14, 15). However, most patients continue to experience either primary or secondary resistance, posing a significant challenge for cancer treatment (16). Developments in immunology, molecular biology, and nanotechnology have significantly redefined cancer treatment and cancer vaccines, which represent a new approach that harnesses the immune system’s ability to identify and eliminate cancer cells (17). However, the clinical outcomes of cancer vaccines are suboptimal primarily due to challenges related to inadequate delivery efficiency, the immunosuppressive tumor microenvironment (TME), and intrinsic resistance (18).

Nanotechnology plays a crucial role in addressing these challenges by providing strategies to overcome the aforementioned limitations through the use of nanocarriers (19). These nanocarriers are designed to enhance the delivery and presentation of tumor antigens, improving the precision and effectiveness of immune activation while specifically targeting lymph nodes, where a significant portion of immune cells can be effectively activated (20, 21). Additionally, by modifying the surface of nanocarriers, it is possible to co-deliver adjuvant molecules that stimulate a robust immune response, directly tackling the issue of tumor-induced immunosuppression (22). Consequently, nanotechnology not only holds great potential for improving the efficacy of current vaccines but also paves the way for the creation of more effective vaccination strategies that are less vulnerable to tumor resistance mechanisms (23, 24). This groundbreaking technology has the capacity to revolutionize cancer immunotherapy, providing treatments that are both more effective and tailored to individual patients (25). In this review, we focus on recent advancements in fundamental immunology and investigates design strategies for developing nanovaccines in the setting of GI cancers. Moreover, we will also examine the ongoing opportunities and challenges associated with the clinical translation of nanovaccines for GI cancer treatment.

## 2 Cancer nanovaccines:mechanisms and types

### 2.1 Mechanisms of cancer nanovaccines

An ideal cancer vaccine is designed to provoke a strong, specific, and long-lasting immune response that effectively addresses the complexities and inherent heterogeneity of cancer (26). Optimal vaccines should not only effectively stimulate the immune system but also be finely tuned to recognize and target the diverse array of tumor-associated antigens found in different cancer cells (27). Nanovaccines signify an advancement over traditional vaccines by leveraging nanotechnology to transform immunization approaches (28). These vaccines employ specially designed nanocarriers to transport tumor antigens derived from tumor cells, viruses, or nucleic acids that encode such antigens (29). Once delivered, these antigens are taken up by antigen-presenting cells (APCs), particularly dendritic cells (DCs), and subsequently presented on major histocompatibility complex (MHC) molecules (30). MHC molecules are encoded by human leukocyte antigen (HLA). HLA class I encodes the first type of MHC molecule, which primarily displays intracellular antigens on the cell surface. This presentation allows CD8^+^ T cells to detect and destroy infected cells. Conversely, HLA class II encodes the second type of MHC, which presents extracellular antigens to CD4^+^ T cells. This interaction promotes the proliferation of T cells and stimulates B cells to generate antibodies that are specific to the presented antigens (31, 32).This mechanism allows T cells to identify the antigens via T cell receptors, resulting in their activation, proliferation, and differentiation into specific effector T cells, which include cytotoxic T lymphocytes (CTLs) and helper T (Th) cells (33, 34). CTLs directly engage and destroy tumor cells using various cytotoxic substances, while Th cells are essential for the clonal expansion of CTLs and facilitate their migration into the TME (35, 36). This collaboration enhances the immune response driven by CTLs, thereby effectively targeting and eliminating tumor cells (36). Adjuvants are vital for enhancing vaccine effectiveness, as they stimulate the activation of APCs and boost the immune response specificity toward the antigens (37). Nanocarriers serve as dual-function vehicles for carrying both antigens and adjuvants, allowing for precise engineering of their surface chemical characteristics (38). This engineering enables targeted release at designated sites, optimizes the release dynamics, and improves the immunogenicity and specificity of the vaccines, ultimately leading to enhanced therapeutic outcomes (39).

### 2.2 Types of nanocarriers

**Figure 1:**
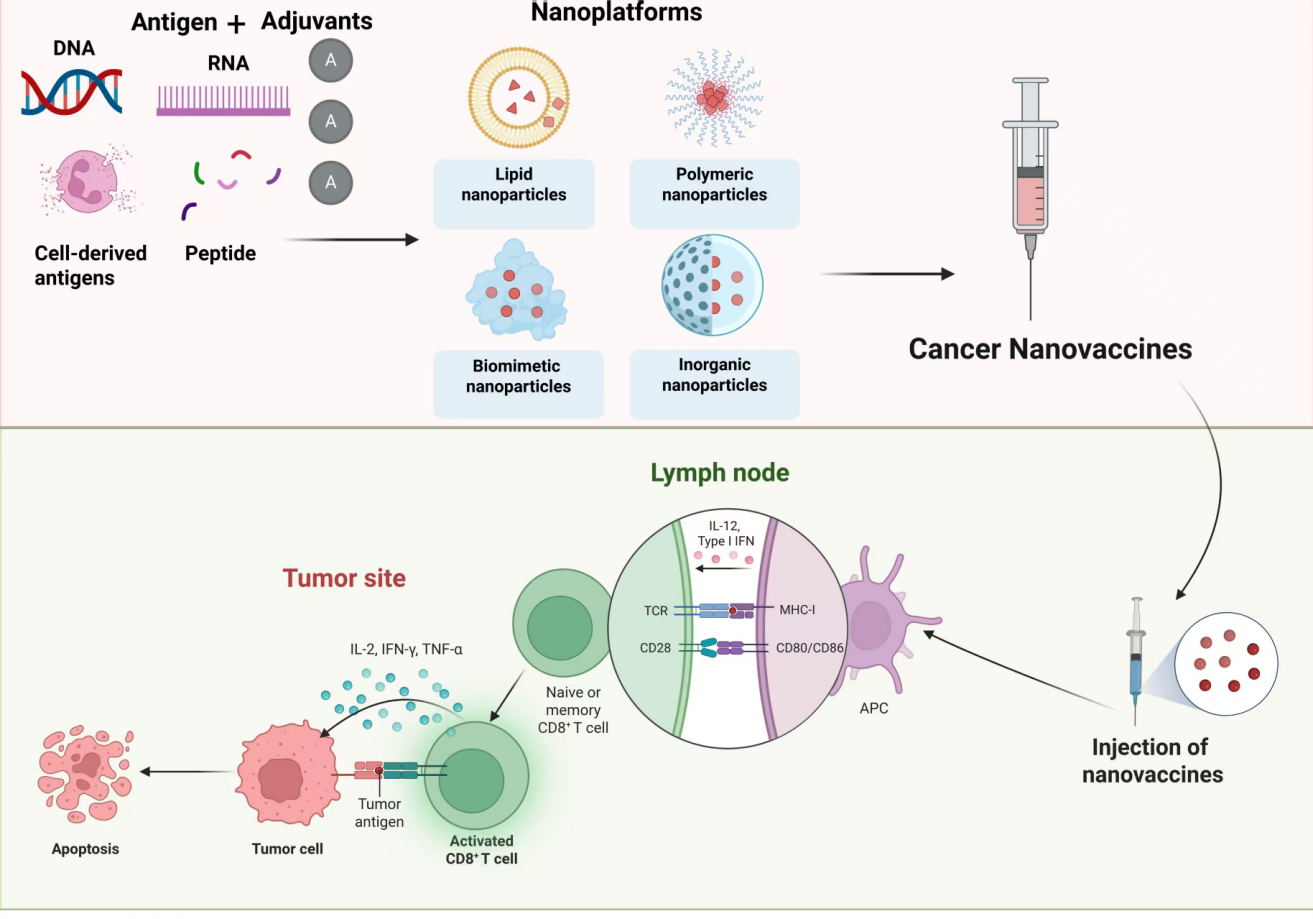
Mechanisms of nanovaccines in gastrointestinal cancer therapy. Various nanoplatforms have been utilized in the formulation of nanovaccines, which can incorporate different types of antigens, including DNA, RNA, and cell-derived peptides, along with adjuvants to enhance immune responses. Once administered, the vaccines stimulate naive or memory CD8^+^ T cells, leading to their activation. Activated CD8^+^ T cells proliferate and migrate to lymph nodes, where they interact with antigen-presenting cells via major histocompatibility complex molecules, promoting a robust antitumor immune response.

A diverse array of nanocarriers has been employed for cancer immunomodulation, which includes lipid nanoparticles (LNPs), polymeric nanoparticles, inorganic nanoparticles, and biomimetic carriers (40) (**Table 1**). As multifunctional delivery platforms, LNPs have demonstrated their effectiveness in encapsulating and transporting various therapeutic agents (41). Recent study has investigated the LNP-based messenger RNA (mRNA) vaccines in cancer immunotherapy. mRNA vaccines enter cells directly through methods such as electroporation to cross the cytoplasmic membrane. Once translated, the protein undergoes modifications and is ultimately taken up and processed by APCs, which degrade it and present the resulting peptides on MHC molecules to T cells, thereby triggering an anti-tumor immune response (42). For example, mRNA-4157 represents a personalized neoantigen therapy utilizing mRNA technology, designed to encode as many as 34 distinct neoantigens and delivered via LNPs. The neoantigens are capable of being translated within the cells, thus activating T cells that specifically target the patient’s tumor neoantigens, providing an innovative strategy for cancer therapy (43). mRNA-4157 could be encapsulated in LNPs, designed to encode up to 34 personalized neoantigens specific to the individual patient (43). And the combination therapy of mRNA-4157 vaccines with pembrolizumab, a humanized antibody that selectively blocked the binding of programmed cell death protein 1 (PD-1) on T cells (44), has demonstrated promising efficacy in patients with resected melanoma (43). In contrast, peptide-based cancer vaccines rely on a robust adaptive immune response to activate their effector functions (45). In recent years, polymeric nanoparticles have garnered significant attention in cancer treatment; poly (lactic-co-glycolic acid) (PLGA), in particular, has shown promise in promoting anti-cancer effects (46). For instance, The NP-TP1@M-M nanovaccine, which was consisted of the TMTP1 peptide and the mannose receptor for DCs assembled on the surface of PLGA nanoparticles, effectively inhibited the growth of ovarian cancer when combined with chemotherapy and ICIs (47). Another intriguing category is inorganic nanocarriers, which demonstrate excellent drug loading capacities but are often susceptible to recognition and phagocytosis by immune cells, presenting a significant barrier to their clinical use (48, 49). Inspired by the “natural camouflage” strategy, biomimetic nanocarriers have emerged as one of the most attractive drug delivery systems (50). These carriers consist of a synthetic nanoscale core that is cloaked in naturally derived cell membranes, leveraging the inherent biological properties of these cells (51). This approach enables homotypic targeting and extends blood circulation time, resulting in increasing applications in biomedicine and targeted drug delivery (50) (**Figure 1**).

**Table 1:** Advantages and disadvantages of different nanocarriers in gastrointestinal cancer vaccination therapy.

Nanocarriers	Advantages	Disadvantages	^Reference^
Lipid nanoparticles	Excellent biocompatibility High drug loading capacity	Complex manufacturing processes	(41)
Polymeric nanoparticles	Effective controlled release Easily functionalized surface	Higher production costs	(46)
Inorganic nanoparticles	High Stability Tunable Optical Properties	Unclear Clearance Mechanisms	(48, 49)
Biomimetic nanoparticles	Superior Biocompatibility and Biodegradability Strong Targeting Capabilities	Complexity in Production	(50, 51)

## 3 Advancements of nanovaccines in GI cancers

### 3.1 Utilizing nanomedicines to treat GI cancers by regulating

#### 3.1.1 cGAS-STING pathway

The cyclic GMP-AMP synthase (cGAS) and stimulator of interferon genes (STING) pathway has become a vital component of the innate immune system (52). Recent advancements in the mechanistic understanding of the STING pathway concerning T cell activation highlight its potential as a promising target for cancer immunotherapy (53). RADA_32_ was a synthetic amphiphilic peptide composed of alternating amino acid sequences, specifically designed to self-assemble into peptide nanofiber hydrogels (54). Huang et al. utilized RADA_32_ peptide hydrogel to encapsulate high-density lipoprotein phospholipid nanoparticles (HPPS), a promising candidate for the platform to form a nanovaccine. It contained antigen peptides and CpG-ODN, called HPPS-AP@RMn, effectively activated the immune response and enhanced recognition and reaction to the antigens. The activation of toll-like receptor (TLR) 9 and cGAS-STING pathways in APCs was crucial for the observed immune response, underscoring the potential of this approach in combating gastric cancer (55). Moreover, Du et al. modified the nanoparticle compositions by adjusting the ingredient ratios, then incorporated sorafenib (SOR) and applied a coating of MIL-100 (Fe) to create MF@SOR that represented a metallic nanovaccine (56). Mechanistically, the MF@SOR bimetallic nanovaccine exhibited responsiveness to specific chemical signals within the TME, triggering pyroptosis and activation of the cGAS-STING signaling pathway. These mechanisms worked synergistically to enhance the maturation of DCs and the infiltration of CD8^+^ T cells, significantly alleviating immunosuppression, which led to the elimination of the primary tumor while generating durable antitumor immune memory, effectively inhibiting tumor progression (56). Radiofrequency ablation is one of the most commonly used minimally invasive techniques for treating hepatocellular carcinoma (HCC), but the presence of residual malignant tissues or small satellite lesions makes complete removal challenging (57). A novel vaccine composed of cyclic GMP-AMP (cGAMP) (a STING agonist) and adsorbed tumor-associated antigens was developed to activate the STING pathway, enhancing the efficacy of immunotherapy and significantly preventing the recurrence and metastasis of HCC (58) (**Table 2**).

**Table 2:** The clinical and preclinical trials of nanovaccines in gastrointestinal cancer therapy.

Nanovaccine	Cancer types	Mechanism	Stage	Reference
PNVAC	Gastric/Gastroesophageal Junction Cancer	Inducing both CD4^+^ and CD8^+^ T cell responses as well as antigen-experienced memory T cell phenotype	Clinical (ChiCTR1800017319)	(82)
Autogene cevumeran	Pancreatic cancer	Inducing a robust neoantigen-specific T-cell response	Clinical (NCT04161755)	(83)
HPPS@RMn hydrogel system	Gastric cancer	Activation of both TLR9 and cGAS-STING pathways	Preclinical	(55)
MF@SOR	Hepatocellular carcinoma	Activation of pyroptosis and the cGAS-STING pathway,which promote dendritic cell (DC) maturation	Preclinical	(56)
LDHs-cGAMP	Hepatocellular carcinoma	Integrating the cancerous antigens released byradiofrequency ablation, which promoted the reinforcement of anti-programmed death ligand 1 immunotherapy.	Preclinical	(58)
MSLN-based nanovaccine	Pancreatic cancer	Eliciting higher humoral and cellular antigen-specific responses	Preclinical	(60)
CCM-PLGA/GA NPs	Colorectal cancer	Activating the maturation of DCs and the formation of a positive anti-tumor immune microenvironment.	Preclinical	(65)
NP-TCL@APS	Colorectal cancer	Promoting the maturation of DC and induce strong responses by T lymphocytes to exert anti-tumor effects.	Preclinical	(67)
LBP-CD155L NVs	Colorectal cancer	Facilitating the endocytosis and maturation of DCs via TLR4 and MGL pathways	Preclinical	(68)
Hypoxia-activated cascade nanovaccine	Hepatocellular carcinoma	Improving immunogenic tumor microenvironment and triggering strong antitumor immune responses by increasing the infiltration of immune cells under hypoxic environment	Preclinical	(69)
TPOP	Colorectal cancer	Capturing tumor antigens and inducing specific recognition by tumor-infiltrating dendritic cells to be taken up.	Preclinical	(73)
MLP-aTIM-3	Colorectal cancer	Providing cognate antigens and costimulation to the exhausted T cells by the T-cell immunoglobulin and mucin domain-containing protein 3 (TIM-3)/aTIM-3 interaction	Preclinical	(74)
LipoFM-CPG	Colorectal cancer	Enhancing the capacity for simultaneous delivery of multiple antigens and adjuvants to dendritic cells, thereby optimizing effective antigen presentation and promoting robust downstream immune responses.	Preclinical	(77)
*E. coli* (AH1-CDA-Co1)@iPDA	Colorectal cancer	*E. coli* (AH1-CDA-Co1)@iPDA could enter Peyer’s patches through M cells, triggering long-term tumor-specific immune memory responses.	Preclinical	(78)
mD@cSMNs	Hepatocellular carcinoma	Polarizing the protumoral N2 phenotype neutrophils to antitumor N1 phenotype for improving the immune effects	Preclinical	(80)

### 3.2 Utilizing nanomedicines to treat GI cancers by remodeling TME

PLGA represents a promising delivery material that has been widely explored in preclinical models. Mesothelin (MSLN) is a tumor-associated antigen that is overexpressed in pancreatic ductal adenocarcinoma (59). The MSLN peptide could be effectively encapsulated within PLGA-chitosan nanoparticles, allowing for subsequent uptake by DCs. The application of MSLN nanovaccination has been shown to effectively inhibit the growth and metastasis of pancreatic tumors, while also significantly increasing the infiltration of CD8^+^ T cells in both preventive and early therapeutic regimens (60). Gambogic acid (GA) plays a role in modulating the tumor immune microenvironment and can be combined with various anti-tumor treatment strategies (61–64). GA functions both as an effective agent that directly targets and kills tumor cells and as an immunoadjuvant that promotes the infiltration of CD3^+^CD8^+^ T cells into tumor tissues. It achieves this by modulating the tumor immune microenvironment and facilitating the maturation of DCs in the draining lymph nodes (65). Based on this premise, Huang and colleagues developed a novel nanovaccine, CCM-PLGA/GA NPs, which was synthesized using GA as an adjuvant in conjunction with neoantigens provided by cancer cells. This formulation demonstrated significant effectiveness in promoting the maturation of DCs and fostering a positive anti-tumor immune microenvironment (65). Astragalus polysaccharide (APS) has the capability to inhibit the migration and invasion of colorectal cancer (CRC) cells. Furthermore, studies indicate that APS modulates immune-active factors to enhance its antitumor effects (66). The nanovaccine (NP-TCL@APS) comprised PLGA nanoparticles encapsulating CRC tumor cell lysates along with astragalus polysaccharides, leading to marked tumor-suppressive effects (67).

Nanovaccines have exerted significant impacts on the regulation of TME and significantly influenced immunotherapy in the setting of GI cancers. Nanovaccine LBP-CD155L NVs, enhanced the endocytosis and maturation of DCs via the synergistic galactose type lectins and TLR4 pathway, which mitigated immune suppressive microenvironments by targeting myeloid-derived suppressor cells and regulatory T cells and exhibited a synergistic effect when combined with anti-PD-1 therapy in CRC (68). The zeolitic imidazolate framework nanoparticles, which carried the hypoxia-activated prodrug tirapazamine and the immune adjuvant resiquimod, facilitated the simple *in situ* formation of a nanovaccine (TRZM). It enhanced the capacity to eliminate HCC cells in hypoxic conditions, also improved the immunogenicity of the TME, thus effectively triggering robust antitumor immune responses by increasing the infiltration of cytotoxic T cells (69). The intestine serves as the largest peripheral immune organ. Its immune system is composed of specialized epithelial cells that create a physical barrier, alongside immune cells found in the lamina propria, a thin layer of connective tissue beneath the epithelium. These immune cells act as the initial line of defense against invading pathogens (70). The enrichment of colibactin, a toxic metabolite produced by Escherichia coli, can promote the advancement of CRC by activating the senescence-associated secretory phenotype (SASP) in malignant or precancerous epithelial cells, suggesting the importance of intestine immune in the development of cancer (71, 72). Researchers have developed a nanovaccine called TPOP, which focused on regulating lipid metabolism and stimulating the innate immune response in the subcutaneous mouse CRC model. Notably, TPOP exhibited significant therapeutic effects in subcutaneous mouse models of CRC and melanoma through decreasing lipid accumulation. Additionally, when used in conjunction with ICIs, TPOP markedly inhibited the growth of distant tumors via systemic anti-tumor immune responses, offering a promising and safe approach to enhancing immune cell function through metabolic manipulation and effective immune system activation (73). By fusing antigen-sensitive DC membranes with TIM-3-targeted LNPs (MLP-aTIM-3), the therapy provided costimulation and specific antigens to exhausted T cells. This approach has demonstrated superior antitumor efficacy in an orthotopic pancreatic cancer model. Furthermore, the therapeutic benefits of MLP-aTIM-3 extended to other tumor models, including liver metastases and CRC (74).

Microbiota play a crucial role in maintaining human health. Certain bacteria that reside in tumors are known to influence tumor growth, metastasis, and responses to various treatment modalities, including chemotherapy, radiotherapy, and immunotherapy (75, 76). Chen et al. integrated highly immunostimulatory adjuvant cholesterol-modified CpG oligonucleotides into autologous membranes derived from *Fusobacterium nucleatum* (*F. nucleatum*). This nanovaccine, which incorporated both bacterial membranes and adjuvants, significantly enhanced the co-delivery of various antigens and adjuvants to DC while reducing cancer metastasis in CRC infected with *F. nucleatum* (77). Additionally, *Escherichia coli (E. coli)* strain (AH1-CDA-Co1) was a genetically engineered strain of *E. coli* designed for oral administration. By coating the bacteria with a polydopamine system (iPDA), the sustained release of engineered outer membrane vesicle (OMV) vaccines was triggered under ultrasound exposure, inducing long-term, antigen-specific immune responses that might hold promise for enhancing immunotherapy in CRC (78). These findings highlight the emerging importance of the host and tumor microbiota in mediating responses to immunotherapy, which await further investigations.

### 3.3 Neoantigen nanovaccines in GI cancers

**Figure 2:**
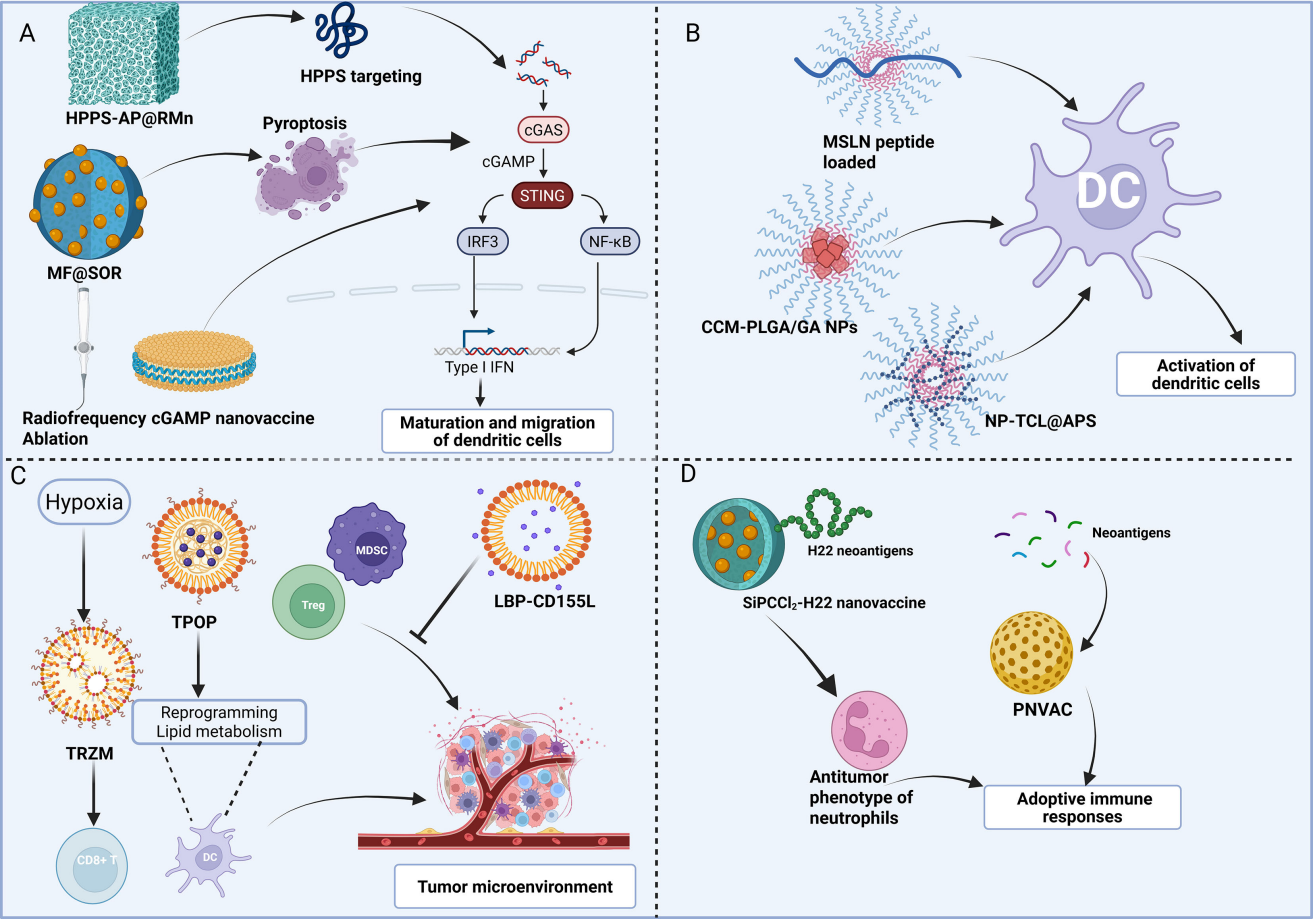
Innovative strategies for nanovaccine-based immunotherapy in gastrointestinal cancers. The figure collectively highlights the multifaceted approaches to modulating the immune system to combat gastrointestinal cancers, emphasizing the critical roles of various nanoparticle systems and signaling pathways.

Neoantigen nanovaccines represented an innovative strategy that offered insights into developing novel immunotherapeutic agents (79). Wang et al. developed nanovaccine that was composed of silicon phthalocyanine dichloride (SiPCCl2)-hybridized mesoporous silica and Fe(III)-captopril, and it was coated with exfoliated membranes of mature DCs stimulated by H22-specific neoantigens. Mesoporous silica embedded with SiPCCl2, designated SMN, served as a nanoscale photosensitizer. Exploiting the material’s intrinsic porosity and coordination chemistry, Fe(III)–captopril complexes were integrated into SMN to generate pH-responsive nanotherapeutics designed to modulate tumor-associated neutrophils (80). The mechanism involved inducing cell death through photodynamic therapy, which promoted the release of tumor-associated antigens and enhanced T cell responses, leading to tumor regression in mouse models (80). Photodynamic therapy is widely regarded as a minimally invasive modality that preferentially targets malignant cells and induces cytotoxic effects. This approach employs a photosensitizer which, upon irradiation with light of an appropriate wavelength, generates reactive oxygen species that trigger cancer cell death (81). Recently, a personalized neoantigen nanovaccine (PNVAC) was generated. The PNVAC intervention demonstrated superior protective efficacy in preventing tumor recurrence and was capable of inducing CD4^+^ and CD8^+^ T cell responses, as well as generating antigen-experienced memory T cell phenotypes. Furthermore, the immune response remained durable and was still evident one-year post-vaccination, offering a safe and feasible strategy for delaying gastric cancer recurrence in the phase I clinical trial (ChiCTR1800017319) (82). Moreover, adjuvant administration of autogene cevumeran, an individualized neoantigen vaccine formulated with uridine mRNA in LNPs together with atezolizumab and chemotherapy appeared to be safe and practicable, and elicited robust neoantigen−specific T−cell responses in approximately 50% of unselected patients with resectable pancreatic cancer (NCT04161755) (83) (**Figure 2**).

## 4 Clinical translation and challenges

### 4.1 Precision delivery and biological barrier challenges

Nanovaccines hold significant promise as a novel approach to cancer immunotherapy; however, the transition from preclinical studies to widespread clinical application faces substantial hurdles. These scientific, technical, and regulatory challenges need to be addressed comprehensively to unlock the full therapeutic potential of nanovaccine technology. One of the primary challenges in advancing nanovaccines lies in achieving efficient and targeted antigen delivery (84). To successfully meet this objective, nanoparticles must effectively navigate various biological barriers, such as the impediments posed by the vascular and endothelial barriers (85, 86). The tight junctions between endothelial cells create a natural blockade that not only affects the distribution of nanoparticles but may also lead to the accumulation or blockage of antigens within the body (87). The size, shape, and surface charge of nanoparticles significantly influence their biodistribution across various organs. Particles larger than 2,000 nm tend to accumulate easily in the spleen and liver, as well as in the pulmonary capillaries. Nanoparticles in the range of 100–200 nm can extravasate through the vascular windows of tumors and evade filtration by the liver and spleen. Conversely, smaller nanoparticles (<5 nm) are filtered out by the kidneys (88). Thus, the structure of nanoparticles play an significant role in drug delivery. Furthermore, the presence of a dense extracellular matrix creates additional physical resistance for nanoparticles as they attempt to traverse tissues, thereby reducing their penetration efficacy within the TME (89). Additionally, immune clearance mechanisms, such as those involving the mononuclear phagocyte system, pose further challenges impacting the effectiveness of nanoparticles (90). These mechanisms can lead to the premature elimination of nanoparticles before they reach their intended tumor sites, ultimately diminishing therapeutic efficacy (91). Consequently, it is essential to explore and develop novel strategies that can efficiently overcome these biological barriers to enhance the efficacy, specificity, and overall therapeutic potential of nanovaccine-based approaches.

### 4.2 Tunable pharmacokinetics

The pharmacokinetic characteristics of nanomedicines are critically important for their efficacy in clinical treatments, encompassing multiple processes such as distribution, metabolism, and excretion within the body (92). These pharmacokinetic properties directly influence the efficiency with which a drug reaches its target and its overall performance in the body (93). To achieve improved therapeutic results, developing nanomedicines with adjustable elimination rates represents a promising strategy (94, 95). These nanomedicines can dynamically modulate their excretion rates in response to changes in the internal environment or the specific needs of target tissues. Such flexibility effectively minimizes drug accumulation in non-target tissues, thereby reducing the risk of adverse effects and providing patients with a safer treatment experience (96–98).

### 4.3 Tumor heterogeneity

The inherent heterogeneity of tumors at genetic and microenvironmental levels poses significant challenges for the effective use of nano-prodrugs in cancer therapy (99, 100). Variations exist not only between individuals but also within different regions of the same tumor, affecting how nano-prodrugs respond to external stimuli such as pH, temperature, or enzyme activity (101–103). This inconsistency in drug release can limit the therapeutic efficacy of nano-prodrugs. Microenvironment-responsive polymer carriers represent an essential strategy that can be effectively utilized for drug delivery, exhibiting high biocompatibility in healthy tissues (104, 105). Upon reaching the TME, these nanoparticles can achieve targeted release, transforming the microenvironment from immunologically insensitive to immunologically sensitive, thus promoting the development of long-lasting immune memory and helping to prevent tumor recurrence (106).

### 4.4 Lack of proper preclinical models

Preclinical models play a vital role in cancer drug discovery; however, commonly used models often fail to accurately reflect the immune biology of human cancers (107, 108). These models rely on the inoculation of cancer cell lines, and the resulting tumors frequently do not replicate the immune environment found in actual tumors. Additionally, they do not account for the gradual accumulation of mutations seen in human cancers, leading to the development of more stable tumors (109). Therefore, there is an urgent need to develop animal models that better mimic the characteristics of human tumors, facilitating the effective translation of preclinical findings into clinical applications. Integrating multiple models can yield more reliable conclusions. Therefore, it is important to emphasize the diversity of models in research to comprehensively assess the effects of new therapies. Implementing such strategies could not only significantly enhance the effectiveness of nanovaccines in cancer treatment but also pave the way for their further development and clinical application.

### 4.5 Screening and identifying neoantigens

Neoantigen-based vaccines deliver personalized mutant peptides or RNA-encoded epitopes directly, thereby expanding the populations of tumor-reactive T cells. By harnessing the precision of adaptive immunity, these vaccines aim to achieve long-lasting anti-tumor responses (110, 111). Unlike traditional personalized therapies that aim to identify specific patient subgroups, these neoantigen-based cancer vaccines are tailored specifically for individual patients (112). Advances in artificial intelligence may provide robust solutions for identifying personalized antigens or developing targeted nano-delivery systems that address the unique characteristics of a patient’s TME (113). Researchers have developed the PISTE algorithm for screening tumor neoantigens. In a prospective study on prostate cancer, 75% of patients exhibited an immune response to neoantigens predicted by PISTE, demonstrating its substantial potential to advance neoantigen-based cancer immunotherapy research (114). Autogene cevumeran was an individualized immunotherapy that utilized a uridine messenger RNA lipoplex. It was specifically tailored to target neoantigens based on data from somatic mutations unique to each patient’s tumor tissue, aiming to elicit T cell responses against as many as 20 different neoantigens (115). It has represented a significant milestone in precision medicine, as researchers employed liposomal technology to deliver neoantigen mRNA vaccine that triggered a multi-antigen specific immune response, which has resulted in a remarkable immune response in patients with advanced or recurrent solid tumors (115). Moreover, *in situ* antigen-capturing nanovaccines represent a promising therapeutic strategy by capturing tumor-derived antigens, thereby reducing immune escape and facilitating the *in situ* formation of antigen libraries. The advantages are primarily achieved through the functional modification of surface groups or the incorporation of synergistic materials, which simplifies the design of nanovaccines and supports the development of lightweight and highly effective cancer vaccines (116). The future advancement of personalized nanovaccines holds even greater potential for cancer patients.

## 5 Conclusions and perspectives

Leveraging their unique advantages, nanotechnology have demonstrated tremendous promise in both the prevention and treatment of GI cancers, offering innovative solutions that could transform current therapeutic approaches (28). DC-based hybrid membrane nanoparticles possess significant potential for improving the effectiveness of ICIs, providing a theoretical basis for enhancing their efficacy in the treatment of Lynch syndrome-associated colorectal cancer (LS-CRC). The nanomedicine delivery system DCsLipo@MnO2@si-CTLA4@PD-1α effectively enhanced T cell proliferation and activation, while also increasing the cytotoxic effects of T cells against cancer cells. Additionally, it played a crucial role in inhibiting the progression of LS-CRC (117). Moreover, Madamsetty et al. loaded irinotecan and curcumin concurrently into ultra-small PEGylated nanoparticles (ND-IRT + CUR), which significantly downregulated IL-10 expression and exhibited notable anti-tumor effects, highlighting the potential application of this nanocarrier in the treatment of pancreatic cancer (118). Additionally, nanomedicine-based cancer vaccine also plays a significant role in GI cancer therapy. This review has delved into the substantial advancements made in the development of nanovaccines targeting GI cancers, which remain one of the most challenging areas in oncology due to factors such as tumor heterogeneity and the complexity of the TME. Despite these advancements, several formidable challenges persist in the journey from laboratory innovations to clinical applications. The process of scaling up manufacturing to meet clinical demands is complicated, often hindered by technical and regulatory hurdles. Additionally, navigating the intricate landscape of obtaining regulatory approvals for new therapeutic modalities is a critical step that requires meticulous planning and collaboration among researchers, manufacturers, and regulatory bodies.

To ensure regulatory compliance and facilitate clinical translation, there is an urgent need to establish a comprehensive and standardized framework for the design, testing, and reporting of nanovaccines. Accelerating the clinical implementation of nanovaccine technologies can be achieved through enhanced collaboration among academia, industry, and regulatory bodies. Fully harnessing the potential of nanovaccines could usher in a new era of innovative and effective treatments for GI cancers, significantly enhancing therapeutic efficacy while also improving the quality of life for patients. These advancements could result in substantial progress in the fight against these malignancies, providing personalized treatment options that are tailored to the unique characteristics of GI cancers.
